# The WHO costing and budgeting tool for national action plans on antimicrobial resistance—a review and assessment of impact in Africa

**DOI:** 10.1093/jacamr/dlag094

**Published:** 2026-05-30

**Authors:** Yidnekachew Degefaw Mazengiya, Laetitia Gahimbare, Ambele Judith, Alessandro Patriarchi, Paul Verboom, Walter Fuller, Yahaya Ali Ahmed

**Affiliations:** Antimicrobial Resistance Unit, World Health Organization, Regional Office for Africa, Brazzaville, Republic of Congo; Antimicrobial Resistance Unit, World Health Organization, Regional Office for Africa, Brazzaville, Republic of Congo; Antimicrobial Resistance Unit, World Health Organization, Regional Office for Africa, Brazzaville, Republic of Congo; AMR National Action Plans and Monitoring Unit, WHO Headquarters, Geneva, Switzerland; AMR National Action Plans and Monitoring Unit, WHO Headquarters, Geneva, Switzerland; Antimicrobial Resistance Unit, World Health Organization, Regional Office for Africa, Brazzaville, Republic of Congo; Antimicrobial Resistance Unit, World Health Organization, Regional Office for Africa, Brazzaville, Republic of Congo

## Abstract

**Background:**

Antimicrobial resistance (AMR) is a global public health crisis affecting human health, animal health, plant health, and the environment. In 2015, all WHO Member States endorsed the Global Action Plan on AMR and committed to develop multi-sectoral national action plans (NAPs) under a ‘One Health’ approach. To accelerate the implementation of AMR NAPs, the WHO introduced a costing and budgeting tool in 2021 to support prioritization of interventions, identification of activities financed through existing budgets, estimation of implementation costs and assessment of funding gaps.

**Objectives:**

To assess the impacts resulting from the use of the costing and budgeting tool at national level in WHO African region (AFRO) Member States.

**Methods:**

National AMR focal points from the human, animal, agriculture and environmental sectors in 15 WHO African Region Member States trained on the tool completed a questionnaire using Google Forms via a secure web-based survey link.

**Results:**

Eleven countries reported mobilizing funding using costed data generated through the tool. Costed NAPs informed policy decisions in seven countries and improved stakeholder collaboration in funding efforts. However, the use of the tool revealed significant challenges, including insufficient domestic financing and lack of congruence of donor priorities with national needs.

**Conclusions:**

Costing One Health AMR NAPs serves as an important catalyst for realistic budgeting and resource planning including resource mobilization to ensure both effective implementation and the long-term sustainability of AMR interventions.

## Introduction

The WHO declared antimicrobial resistance (AMR) as one of the top 10 global health priorities.^1^ AMR is a growing threat to human, animal, environmental health and food production.^2–4^ It compromises global health security, achieving universal health coverage and the achievement of Sustainable Development Goals.^5^ In response to the threat of AMR, all WHO Member States endorsed the 2015 Global Action Plan on AMR and committed to develop multi-sectoral national action plans (NAPs) under a ‘One Health’ approach.^6^ In the WHO African Region (AFRO), all 47 Member States have developed multi-sectoral NAPs as of 2025.^7^

In September 2024, the United Nations General Assembly adopted a political declaration on AMR, underscoring the urgent need for global action to combat AMR, which is responsible for ∼4.95 million human deaths annually. The declaration underscores the importance of costing NAPs to effectively address AMR and emphasizes the need to quantify and allocate financial requirements to ensure sustainable implementation of NAPs across key AMR interventions.^8^ While progress has been made, the implementation of NAPs for AMR in African countries remains at varying stages. In 2024, findings from Global Database for Tracking Antimicrobial Resistance (AMR) Country Self-Assessment Survey revealed that, out of 47 countries in AFRO that developed NAPs, 35 countries (74%) reported their NAP approved by government and is being implemented and 46% had a costed and budgeted operational plan.^9^ Some of the key reasons explaining the discrepancy between NAP development and implementation are low country-level prioritization, absence of costed plan, insufficient technical capacity and limited funding to follow through on planned and prioritized activities.

Understanding the cost of implementing AMR NAPs is critical for effectively integrating these activities into national health budgets as part of the health system strengthening. With the exception of South Africa, most countries in Africa have not yet met the commitment of allocating at least 15% of their national budgets to health, as outlined in the 2001 Abuja Declaration.^10^ As a result, overall health sector funding remains limited, and resources are often stretched across competing priorities. Given the relatively small size of the health budget envelope in these settings, mobilizing dedicated funding for AMR NAP implementation remains necessary.

Effective and sustainable implementation of NAPs necessitates that key interventions are prioritized, practical and realistic, along with detailed costing and budgeting of these operational plans and securing the financing towards the costed plans.^11^ Having a costed NAP is imperative as it provides decision makers with insights into the costs of activities, what is funded, identifies funding gaps and enables the leveraging of additional resources. However, in many cases, AMR NAPs remain aspirational, and the costs are not integrated into other existing national plans or budgets and therefore implementation is not sustainable.

Cognizant of these needs, WHO launched a costing and budgeting tool for AMR NAPs in October 2021 to assist countries.^12^ The tool was developed over 18 months on the basis of extensive consultations with internal and external experts, review of existing tools and their deficiencies and feedback from potential users in countries. It is a Microsoft Excel-based tool that guides users through the process of inputting cost and financing components, and inflation and currency assumptions, to combine and compare cost and budgeting estimates over a 1–5-year period.^13^ This tool serves as a valuable resource for countries to prioritize activities, generate detailed budget of AMR NAPs and to generate meaningful data to support financing decisions and enhance resource mobilization including linking with broader system-strengthening initiatives or sector-wide plans funded by the national budget or other partners. The tool is pragmatic, modular, user-friendly and adaptable to the specific needs of countries to generate detailed costs of their AMR NAP activities and has also been used to cost other plans. This study aimed to assess the impacts resulting from the use of the costing and budgeting tool at national level in WHO African region (AFRO) Member States. Specifically, the study assessed (i) the extent to which the costing and budgeting tool supported resource mobilization for AMR NAP implementation, (ii) its influence on policy decision-making and prioritization of AMR interventions, (iii) its contribution to stakeholder coordination across One Health sectors and (iv) challenges encountered in mobilizing resources using costed NAPs. In this study, ‘impact’ refers to the reported effects of using the WHO costing and budgeting tool to support the costing of AMR NAPs and its influence on resource mobilization, policy prioritization, stakeholder coordination and decision making.

### Use of the WHO costing and budgeting tool in the AFRO region

Sierra Leone was the first country to pilot test the prototype tool. Following the nomination of 12 costing coordinators from key ministries, a 3-day training was conducted in January 2021 using a hybrid (virtual and in-person) mode due to COVID-19 restrictions, resulting in a fully costed 2-year operational plan for its NAP on AMR. The lessons learned from the pilot along with additional pilots in Paraguay, Jamaica and Somalia were instrumental in finalizing the tool, which was launched in October 2021.^14^ Following these pilots, the three levels of WHO (Headquarters, the Regional Office for Africa and the WHO Country Office) in collaboration with partners, supported countries to build their national capacity on the use of the tool.

Support to countries was provided through dedicated training/workshops to share the Microsoft Excel-based tool, ensure transfer of knowledge and practical know-how on how to use the tool. As part of the first batch of training that ran from 2021 to 2023, over 260 professionals from human, animal, agriculture and environmental sectors across 15 countries, along with representatives from four partner organizations [Africa CDC, FAO, ReAct Africa and World Organization for Animal Health (WOAH)] were trained on the use of the tool. These 15 countries, representing 32% of WHO African Region Member States, were selected on the basis of their participation in the training initiative and readiness to develop costed AMR NAPs. After the training, each country developed costed versions of their national plans. The total cost estimates for NAP implementation in trained countries ranged from USD 1.2 million to USD 77.6 million, showing a huge variation in scope of activities and contextual priorities.^15^ Of the 15 participating countries, seven are low-income country (LIC) as per the World Bank income group classification of 2024, while eight belong to MIC (Table 1).^16^

**Table 1.** List of AFRO countries trained on the use of WHO costing and budgeting tool for AMR National Action Plans and their AMR NAP costs

S/No.	Country	World Bank income group	NAP implementation timeframe	Total cost (USD)
1.	Burundi	LIC	2020–2023	6 245 244
2.	Central African Republic	LIC	2024–2028	Pending endorsement by the national authority
3.	Comoros	MIC (lower)	2022–2026	3 367 314
4.	The Gambia	LIC	2023–2027	1 293 502
5.	Equatorial Guinea	MIC (upper)	2023–2027	Pending endorsement by the national authority
6.	Kenya	MIC (lower)	2023–2027	15 759 303
7.	Mauritius	MIC (upper)	2023–2027	4 310 143
8.	Nigeria	MIC (lower)	2024–2028	77 633 889
9.	Rwanda	LIC	2025–2029	29 585 716
10.	Sao Tome and Principe	MIC (lower)	2023–2025	1 333 100
11.	Sierra Leone	LIC	2018–2022	2 146 000 (only a 2-year prioritized operational plan was costed)
12.	South Sudan	LIC	2023–2028	11 818 591
13.	United Republic of Tanzania	MIC (lower)	2023–2028	25 435 052
14.	Zambia	MIC (lower)	2023–2027	27 506 417
15.	Zimbabwe	LIC	2024–2028	45 101 429

## Methods

In early 2024, the AMR Unit/WHO AFRO developed an online questionnaire to assess the impact of the use of costed AMR NAPs in facilitating resource mobilization, assess their influence on policy decisions, stakeholder engagement and identify challenges encountered in mobilizing resources. The questionnaire was developed based on the objectives of the study and included both closed and open-ended questions. Closed-ended questions were primarily structured as yes/no and multiple-choice response options, while open-ended questions allowed respondents to provide free-text responses. Quantitative data were summarized descriptively, while qualitative data from the open-ended questions were reviewed and coded, and similar responses were grouped into common categories using thematic content analysis. Content validity was ensured through multiple rounds of review by technical experts within the study team and external reviewers from WHO Headquarters. However, the questionnaire was not pilot tested prior to implementation. It was translated into three languages: English, French and Portuguese. The questionnaire was administered using Google Forms via a secure web-based link. This approach is consistent with previous regional implementation assessments of antimicrobial resistance NAPs in the WHO African Region.^17^

The survey was distributed to a diverse group of respondents across 15 countries. These countries were selected using purposive sampling, based on their participation in WHO-led training sessions on the costing and budgeting tool and their subsequent use of the tool to cost their AMR NAPs. The sample size of 15 countries reflects the total number of eligible WHO AFRO Member States that had completed both the training and the costing process during the study period, ensuring full coverage of the target population for this assessment.

Participants were eligible for inclusion if they were sector-specific national AMR focal points from the human health, animal and agriculture, or environment sectors with direct involvement in the costing and implementation of their country’s AMR NAP. In addition, partners supporting the implementation of AMR NAPs at the country level, including representatives from WHO country offices, Africa CDC, FAO, ReAct Africa and WOAH, were included. All participants were validated for relevance by the national AMR focal person through the WHO Country Office. Exclusion criteria applied to individuals who were not available during the data collection period, had not participated in the training or whose participation was not endorsed by the national AMR focal person. Data were collected from 6 June 2024 to 8 July 2024, and extracted into Microsoft Excel for analysis.

### Ethics

This study did not involve the collection or analysis of patient-level data. Consent was provided by participants on completion of questionnaires. No financial incentives were provided for participation.

## Results

The survey received responses from 12 of the 15 targeted countries, as well as two of the four partner organizations. All responses from these countries and partner organizations were included in the analysis. The survey captured response from national AMR focal points, with some countries contributing responses from multiple sectors, while others had representation from a single sector or WHO Country Office. The following countries and their respective focal points responded to the survey: Burundi, Central African Republic, Comoros, Equatorial Guinea, Kenya, Mauritius, Nigeria, Rwanda, Sierra Leone, South Sudan, United Republic of Tanzania and Zambia. A total of 22 responses were received from these countries, representing key sectors involved in the implementation of costed AMR NAPs. Most responses (nine) came from the human health sector, followed by five from the agriculture/animal health sector and three from the environmental sector, reflecting engagement across the One Health sectors. In addition, three responses were received from WHO country offices, and two from partner organizations (React Africa and WOAH).

### Leveraging costed AMR NAPs for resource mobilization

Among the surveyed countries, 11 countries secured funding from the national government and external partners for AMR interventions through at least one ministry, using costed data from their national action plan. These countries include Burundi, Central African Republic, Comoros, Kenya, Mauritius, Nigeria, Rwanda, Sierra Leone, South Sudan, United Republic of Tanzania and Zambia. The amount of funding obtained varied significantly across countries, with the highest reported funding being $5.2 million, followed by $1.1 million, while the lowest was $10 000. However, some countries did not provide specific details regarding the amount of funding secured. As such, the available data provides indicative ranges rather than a comprehensive quantification of funding mobilized across all countries. These findings show both the effort made by countries to use costed data from NAPs for resource mobilization while also revealing disparities in country capacities to leverage costed data to secure financial support from both domestic and external sources.

Furthermore, 10 countries (Burundi, Central African Republic, Comoros, Kenya, Mauritius, Nigeria, Rwanda, Sierra Leone, South Sudan and United Republic of Tanzania) reported using costed data to develop funding proposals for AMR-related projects and programmes, with support from WHO and other partner organizations. These proposals were directed towards various funding sources, including the Global Fund, the Pandemic Fund, the National Action Plan for Health Security, Fleming Fund Grants, USAID and government finance departments, among others. These efforts were reported to support the mobilization of funds for the implementation of priority activities in their respective NAPs.

### Using costed NAPs for strategic decision making and prioritization

Findings from country responses show that seven countries (Burundi, Equatorial Guinea, Mauritius, Nigeria, Rwanda, South Sudan and Zambia) highlighted the contribution of costed AMR NAPs in shaping their policy decisions and prioritization of interventions. The availability of detailed costed data was reported to support more effective engagement with partners and donor agencies, aligning their funding requests with the specific activities outlined in the NAPs. In addition, it encouraged stakeholders to explore the feasibility of securing domestic funding sources for integrating AMR interventions into national sector-specific plans and budgets. The costed data also facilitated lobbying efforts for fund allocation, providing a clear, evidence-based justification for financial needs.

### Costed NAPs to foster coordination of funding efforts among stakeholders

Effective stakeholder coordination is critical as it ensures the alignment of efforts, optimizes resource allocation and enhances the impact of multi-sectoral engagement. Analysis of country responses indicates that seven countries (Burundi, Central African Republic, Equatorial Guinea, Nigeria, Rwanda, South Sudan and Zambia) have observed improvements in the coordination of resource mobilization efforts among various stakeholders following the costing of their AMR National Action Plan. These improvements include the engagement of the environmental sector, which began allocating funding for joint activities; partners are encouraged to pay attention to other sectors that are poorly funded, and AMR focal points are advocating for the inclusion of AMR in sector-specific annual operational plans and budgets. For example, qualitative responses from some countries indicated that increased involvement of the environmental sector in costing process and NAP implementation was reported to support coordinated resource mobilization, including contributions to joint activities and greater inclusion of environmental priorities in funding proposals. In addition, joint proposal development has helped avoid duplication, and regular meetings of partners and donors under the leadership of the national focal point for AMR have enhanced overall coordination.

Furthermore, analysis of responses indicated that most feedback from partner organizations aligns with that from national focal points, highlighting that the cooperation and coordination of funding efforts among stakeholders at the country level might have improved following the costing of AMR NAPs. Country responses on the use of costed AMR NAPs for resource mobilization, decision making, prioritization and stakeholder collaboration are summarized in Table 2.

**Table 2.** Summary of country responses on the use of costed AMR NAPs

S/No.	Country	Utilized costed NAP to secure domestic and external funding	Utilized costed NAP to develop funding proposals for AMR-related projects or programmes	Utilized costed NAP for strategic decision-making and prioritization	Utilized costed NAPs to foster coordination of funding efforts among stakeholders
1.	Burundi	Yes	Yes	Yes	Yes
2.	Central African Republic	Yes	Yes	No	Yes
3.	Comoros	Yes	Yes	No	No
4.	Equatorial Guinea	No	No	Yes	Yes
5.	Kenya	Yes	Yes	No	No
6.	Mauritius	Yes	Yes	Yes	No
7.	Nigeria	Yes	Yes	Yes	Yes
8.	Rwanda	Yes	Yes	Yes	Yes
9.	Sierra Leone	Yes	Yes	No	No
10.	South Sudan	Yes	Yes	Yes	Yes
11.	United Republic of Tanzania	Yes	Yes	No	No
12.	Zambia	Yes	No	Yes	Yes

### Challenges in resource mobilization through costed NAPs

Despite the development of costed AMR NAPs, resource mobilization to implement and monitor these plans remains a significant challenge, as reported by all 12 responding countries (Burundi, Central African Republic, Comoros, Equatorial Guinea, Kenya, Mauritius, Nigeria, Rwanda, Sierra Leone, South Sudan, United Republic of Tanzania and Zambia). The key challenges in resource mobilization identified by these countries can be broadly categorized into three main areas: funding and budget constraints, stakeholder and donor coordination, and policy and administrative barriers (Table 3). Addressing these challenges requires increased domestic investment to ensure sustainable financing for AMR interventions, including through mainstreaming AMR into broader national strategies, strengthened multi-sectoral collaboration and strategic engagement with international partners.

**Table 3.** Challenges in resource mobilization for costed AMR NAPs

Category	Challenges Identified
Funding and budget constraints	Budget allocation without disbursement, insufficient domestic funding to incorporate AMR interventions among other priorities, limited donor interest in supporting specific AMR interventions (earmarked funds) and near-expiry NAPs hindering resource mobilization.
Stakeholder and partner coordination	Weak multi-sectoral coordination, insufficient partner coordination platforms, weak engagement of stakeholders in proposal development, inconsistent donor priorities, limited stakeholder awareness due to the lack of dissemination of costed AMR plans.
Policy and administrative barriers	Lack of government commitment due to absence of policy documents, delays in launching of costed NAPs, misalignment with national budgeting cycles, weak monitoring mechanism, absence of partner mapping documents.

## Discussion

Resource mobilization is crucial for the successful implementation of AMR NAPs. It is one of the six steps recommended by WHO in its AMR NAP implementation handbook.^11^ At present, there is a significant opportunity for countries to mobilize resources to combat AMR through both domestic and external funding mechanisms, including the AMR Multi-Partner Trust Fund, the Fleming Fund, the Global Fund and the Pandemic Fund, offer financial support for implementing AMR NAPs.^18–20^ These external funding opportunities enable national governments to accelerate progress in mitigating AMR. In addition, costed AMR NAPs are a first step towards exploring the feasibility of using domestic resources to finance specific activities through existing national plans and budgets. Our findings demonstrate that nine countries used costed NAP data in developing funding proposals for AMR-related projects targeting global funding sources; this was reported to support WHO country offices in Burundi, Nigeria and Sierra Leone securing funds from the Fleming Fund and the Global Fund. This highlights the importance of the tool in enhancing countries’ capacities to participate in international funding mechanisms.

Moreover, our results indicate that among the surveyed countries, 10 were able to secure funding from government and external partners for AMR interventions. This is consistent with evidence showing that structured financial data strengthens resource mobilization efforts by improving transparency and accountability in funding proposals.^21^ However, the significant variation in the amounts of funding secured across the study countries reflects differences in country capacities to attract financial resources. While some countries achieved substantial funding, others were less successful, indicating a need for further support and financial advocacy, especially in countries with lower funding outcomes.

Detailed implementation costs associated with specific activities in AMR NAPs enables governments to assess feasibility, allocate resources efficiently and identify funding gaps.^13^ The results of this study indicate that seven countries have used costed AMR NAPs to support decision making. This finding aligns with previous research highlighting the importance of financial data in guiding health policy decisions, ensuring that resources are directed towards cost-effective and high impact interventions.^22,23^ Moreover, these countries leveraged costed NAPs to advocate for the integration of AMR interventions into national budgets. This supports studies suggesting that incorporating different programmes into domestic financing frameworks enhances programme sustainability and reduces dependence on external donors.^24^

The findings from this study align with the broader recognition by the Quadripartite organizations that effective multi-sectoral coordination is essential for strengthening One Health governance and addressing AMR across sectors.^25^ The costing of NAPs has played a key role in enhancing collaboration among stakeholders, resulting from the joint prioritization of activities and the actual joint costing exercise. This is evidenced by the seven countries that reported improved coordination of stakeholders for funding efforts following the costing process. This finding is consistent with published case studies from the United Republic of Tanzania and South Sudan, which show that the costing of NAPs catalysed collaboration in these countries.^26,27^

Another key finding from the study is that costed NAPs have strengthened the engagement of sectors that were previously lagging, such as the environment sector, which has now begun mobilizing resources for AMR-related activities. This increased engagement can be linked to the involvement of all sectors in the costing process, as well as the inclusion of environmental priorities in the costed plans, which supported greater ownership and made sector roles more visible in AMR implementation. This aligns with global recommendations emphasizing the need for a holistic, One Health approach to AMR funding, ensuring that all sectors are adequately funded.^8^ The study also revealed that involving different sectors in the costing of NAPs and joint proposal development helped countries avoid duplication of efforts. This is in line with the study indicating that financial transparency and joint planning reduce duplication and optimize the impact of available resources.^24^

Resource mobilization for AMR faces several challenges, particularly in low- and middle-income countries.^28^ A report by WHO highlights that incorporating AMR initiatives into existing programmes and national budgets is essential for sustainability; however, this process is time-consuming, requires much stronger collaboration with other system-strengthening initiatives in the country, and requires highlighting the co-benefits of AMR interventions which might otherwise lead to greater competition for scarce existing resources within the national budgets for various sectors. To maintain focus and commitment to address relatively new and complex challenges such as AMR, and to expedite the implementation of activities, many countries remain heavily reliant on donor funding and international development agencies.^29^ Although costed NAPs have been developed, challenges remain in securing sufficient funding, as reported by all 12 surveyed countries. One of the key barriers identified is insufficient domestic funding. The majority of countries continue to rely heavily on donor funding, which, while crucial, is often subject to volatility, leading to inconsistencies in NAP implementation. As such, incorporating AMR funding into national development strategies and exploring alternative financing mechanisms, such as public–private partnerships and domestic revenue mobilization is critical.^30^ In the context of the human health sector, AMR teams will need to engage closely with the health financing teams to better understand the opportunities that exist for mainstreaming relevant AMR interventions into national health systems strengthening or pandemic preparedness plans and budgets. AMR technical teams and policy makers will also need to be better informed about core aspects of health financing, including revenue raising, pooling of resources, purchasing of health services and designing of benefit packages. WHO is currently piloting in four countries the mainstreaming of the 13 core AMR interventions of its people-centred approach to addressing AMR into primary healthcare-oriented health systems.^31^ Future efforts to secure domestic financing for AMR NAP activities in human health will benefit from the results of this pilot.

Beyond financial constraints, countries reported weak stakeholder coordination and policy, and administrative barriers further delay efforts to mobilize resources effectively. In addition, declining financing for global development efforts puts significant strain on national budgets, and results in intense competition among various initiatives for scarce resources. Studies indicate that strengthening governance mechanisms and advocating for high-level political commitment are critical steps towards securing long-term financial support.^32^

### Limitations

The findings of this study should be interpreted in the context of a few limitations. There is a risk of self-report bias, considering that the AMR focal points subjectively completed the questionnaire, may have reduced the validity of the findings. In addition, as respondents were directly involved in the implementation of costed AMR NAPs, there is a potential for social desirability bias. There was also an imbalance in sectoral representation among respondents, with relatively fewer responses from the environmental sector compared with the human and animal health sectors, which may have limited the breadth of perspectives captured. Furthermore, reported responses were not independently validated against external sources such as national AMR NAP documents or implementation reports, and findings are based on self-reported information without triangulation. No formal power analysis conducted to determine the sample size. The study included all WHO AFRO Member States that had received training and costed their AMR NAPs using the WHO tool. While this approach ensured comprehensive coverage of available cases, it may limit the generalizability of the findings to countries that have not yet adopted or used the tool. Although the questionnaire was refined through multiple expert reviews, it was not pilot-tested on a subset of participants prior to dissemination. This may affect the reproducibility of the tool and the generalizability of the findings.

### Conclusions

The development of costed AMR NAPs was found to support policy decisions, prioritization, strengthening financial planning and enhancing stakeholder engagement. These plans have also facilitated coordination of funding efforts among key actors. However, variations in funding success highlight the need for targeted support to assist countries with lower financial outcomes. Persistent challenges, particularly insufficient domestic funding, underscore the need for stronger advocacy and increased national investment. To further strengthen the progress made, it is crucial for stakeholders and partners to continue supporting the use of the WHO costing and budgeting tool as a joint resource to support all sectors while updating AMR NAPs. In addition, ongoing follow-up on the outcomes of the costing exercise is essential to ensure that identified challenges are gradually addressed.
